# Low serum albumin concentrations are associated with disease severity in patients with myasthenia gravis

**DOI:** 10.1097/MD.0000000000005000

**Published:** 2016-09-30

**Authors:** Yi-Yun Weng, De-Hao Yang, Mei-Zi Qian, Mao-Mao Wei, Fang Yin, Jia Li, Xiang Li, Ying Chen, Zhang-Na Ding, Yi-Bo He, Xu Zhang

**Affiliations:** a Department of Neurology, the First Affiliated Hospital of Wenzhou Medical University; b Department of Endocrinology, the Third Affiliated Hospital of the Wenzhou Medical University; c Department of Anesthesiology, the First Affiliated Hospital of Wenzhou Medical University; d School of the First Clinical Medical Sciences, Wenzhou Medical University, Wenzhou, China.

**Keywords:** disease severity, myasthenia gravis, serum albumin

## Abstract

Serum albumin (S-Alb) is a widely used biomarker of nutritional status and disease severity in patients with autoimmune diseases. We investigated the correlation between S-Alb and the severity of myasthenia gravis (MG).

A total number of 166 subjects were recruited in the study. Subjects were divided into 3 groups (T1 to T3) by S-Alb levels: T1: 21.1 to 38.4 g/L, T2: 38.5 to 41.5 g/L, T3: 41.6 to 48.9 g/L. Regression analysis was performed to determine the correlation of initial albumin concentrations and the severity of disease of MG.

Lower levels of S-Alb were observed in subjects with increased disease severity than those with slight disease severity, meanwhile, incidence of myasthenia crisis increased in the lower albumin tertiles (*P* < 0.001). The disease severity assessment was performed according to the criteria established by the Myasthenia Gravis Foundation of America. After adjusting for age, sex, body mass index (BMI), and duration of disease, it showed that higher S-Alb concentrations were associated with lower disease severity. Odds ratios (ORs) of T2 to T3 were 0.241 (95% CI: 0.103–0.566, *P* < 0.001), 0.140 (95% CI: 0.054–0.367, *P* < 0.001) when compared with subjects in the T1, respectively. When subjects were stratified into hypoalbuminemia and normal albumin groups, we found that the association between S-Alb and MG remained significant in the hypoalbuminemia group only (OR: 0.693, 95% CI: 0.550–0.874, *P* = 0.002) after further adjustment for age, sex, BMI, and duration of disease.

This is the first study to demonstrate that S-Alb was independently associated with MG severity. In patients with low S-Alb, S-Alb concentration could be a potential biomarker for MG disability.

## 1 Introduction

Serum albumin (S-Alb), a predominant product of hepatic protein synthesis, is the major determinant of colloid osmotic pressure and a main plasma carrier of many endogenous and exogenous compounds.^[1]^ This multifunctional protein is also considered as a measure of inflammation and nutritional status, and is usually used for the assessment of inflammatory conditions.^[2,3]^ Recently, several studies confirmed that S-Alb has antioxidant properties and plays a major antioxidant role in extracellular fluids, accounting for approximately 70% of the serum antioxidant capacity.^[4,5]^ Furthermore, S-Alb is already considered an attractive biomarker in many autoimmune diseases, such as rheumatoid arthritis, and graft-versus-host disease.^[6,7]^ As increasing evidence has implicated inflammation and oxidative stress in the immunopathogenesis of myasthenia gravis (MG),^[8,9]^ S-Alb may be associated with the development of MG. Therefore, the aim of this study was to investigate the correlation between the S-Alb and MG, and the relationship between levels of S-Alb and clinical parameters representing severity of disease.

## 2 Materials and methods

### 2.1 Study population

The study enrolled a total number of 166 MG inpatients from the First Affiliated Hospital of Wenzhou Medical University between 2009 and 2015. MG was diagnosed in accordance with standard clinical criteria of characteristic weakness, fatigue, electrophysiology, neostigmine test and/or the presence of autoantibody against skeletal muscle acetylcholine receptors (AChRs).^[10]^ Subjects were excluded if they had chronic liver or kidney diseases, severe infectious diseases, and cancer.

The research protocol of the study was approved by the Ethics Committee of the First Affiliated Hospital of Wenzhou Medical University. Written informed consent was waived because of the retrospective nature of this study. All the records of patients were anonymized and deidentified before analysis.

### 2.2 Clinical and laboratory measurements

At admission, demographic data and medical history were recorded. The disease severity assessment was performed according to the criteria established by the Myasthenia Gravis Foundation of America (MGFA) clinical classification as follows: I = any ocular muscle weakness; IIa = predominantly affecting limb, axial muscles, or both; IIb = predominantly affecting oropharyngeal, respiratory muscles, or both; IIIa = predominantly affecting limb, axial muscles, or both; IIIb = predominantly affecting oropharyngeal, respiratory muscles, or both; IVa = predominantly affecting limb and/or axial muscles; IVb = predominantly affecting oropharyngeal, respiratory muscles, or both; V = defined by intubation, with or without mechanical ventilation, except when employed during routine postoperative management. Thymus histology was presented by means of magnetic resonance imaging (MRI) or computed tomography (CT). As an index of body fat, body mass index (BMI) was calculated as the ratio of weight (kg) to height (m^2^). Blood samples were drawn by venipuncture in the morning after an overnight fast for at least 8 hours. Biochemical parameters were measured using a Clinical Analyzer Beckman Coulter AU5831 (Beckman Coulter, CA, USA), including S-Alb, globulin, total bilirubin (Tbil), indirect bilirubin (Ibil), uric acid, creatinine, and high-density lipoprotein cholesterol (HDL-C). Individuals were classified into 2 groups as “Normal albumin” or “Hypoalbuminemia”, which were defined as S-Alb ≥ 40 g/L and S-Alb < 40 g/L, respectively.

### 2.3 Statistical analysis

All subjects were classified into 3 groups by the S-Alb level, in order to derive a deeper understanding of the relationship between S-Alb level and MG severity. S-Alb was categorized as follows: T1 21.2 to 38.4 g/L, T2 38.5 to 41.5 g/L, T3 41.6 to 48.9 g/L.

The statistical software Statistical Program for Social Sciences version 20.0 (SPSS, Inc., Chicago, IL) was used for all analyses. For continuous variables, results were summarized as mean ± standard deviation (SD), and the differences among groups were analyzed by a 1-way analysis of variance (ANOVA) or Kruskal–Wallis test. Categorical variables were presented as counts or percentages, and intergroup comparisons were analyzed by Chi-squared test. Spearman rank correlation was used to determine the relationship between S-Alb and uric acid. In order to explain the contribution of the variance in MG severity, multivariable models were used for multivariate analysis, including sex, age, duration of disease, diabetes, hypertension, cardiopulmonary disease, globulin, white blood cell count (WBC), Tbil, Ibil, uric acid creatinine, and HDL-C.

## 3 Result

### 3.1 Clinical characteristics

One hundred and sixty-six patients with MG were enrolled in this study. The basic demographic and clinical characteristics of the study subjects by distinct tertiles of S-Alb are summarized in Table 1. Briefly, there were no differences in the age of the enrolled subjects and the proportions of men and women among the 3 groups.

**Table 1:**
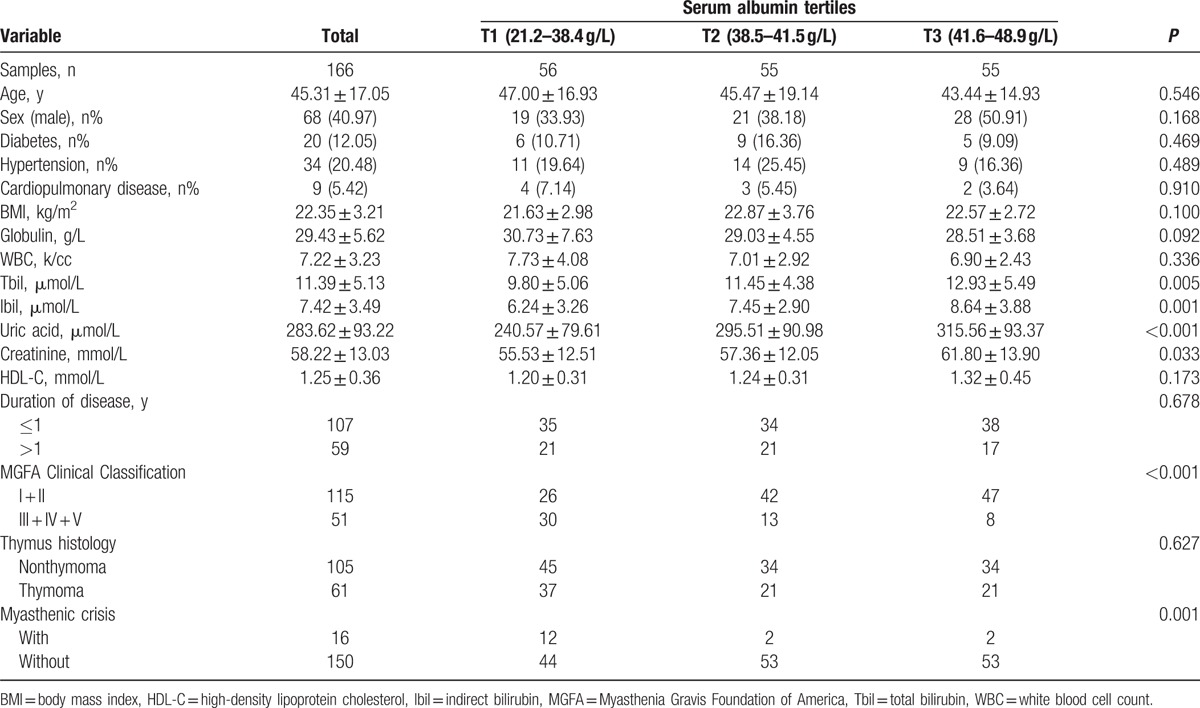
Characteristics of the 166 participants according to the tertiles of serum albumin.

Tbil, Ibil, uric acid, creatinine tested were significantly different among the 3 tertiles (*P* < 0.05). As expected, patients with lower S-Alb had higher MG severity and higher rate of incidence of myasthenic crisis (*P* < 0.05).

### 3.2 Association of S-Alb with MG severity

**Figure 1:**
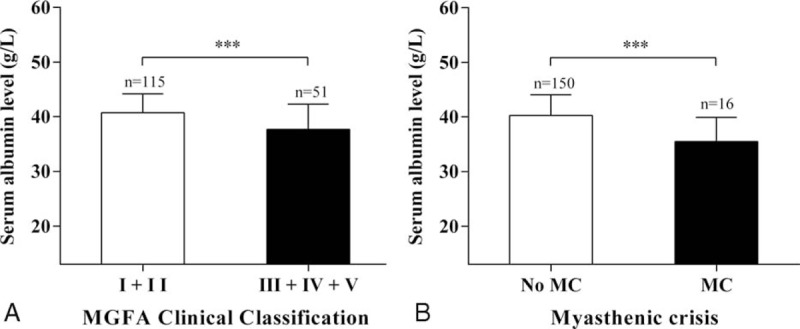
Correlation between S-Alb and other clinical factors in MG patients. (A) Relationship between S-Alb and 2 subgroups according to the MGFA Clinical Classification (^∗∗∗^*P* < 0.001). (B) Relationship between S-Alb and MC (^∗∗∗^*P* < 0.001). MC = myasthenic crisis, MG = myasthenia gravis, MGFA = Myasthenia Gravis Foundation of America, S-Alb = serum albumin.

**Figure 2:**
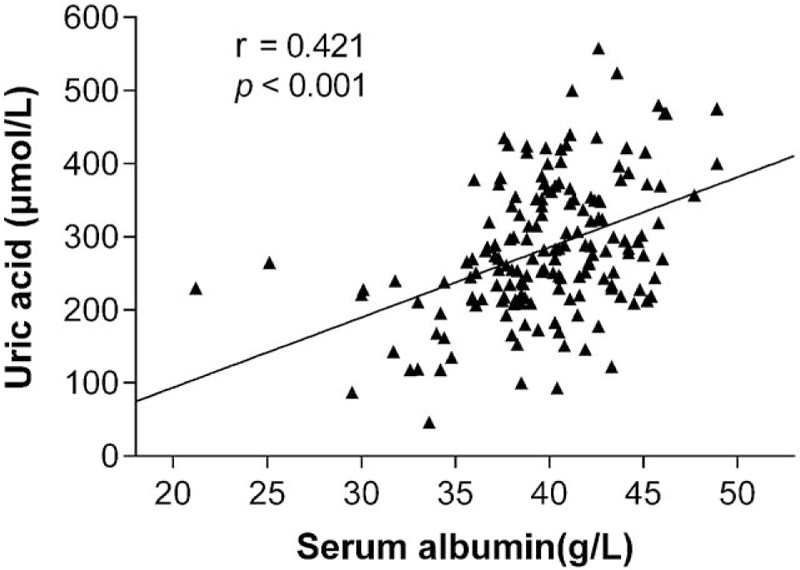
Relationship between S-Alb and uric acid in patients with MG. S-Alb was well correlated with uric acid (*r* = 0.421, *P* < 0.001). MG = myasthenia gravis, S-Alb = serum albumin.

As shown in Fig. 1A, patients with higher disease severity had a lower S-Alb than those with lower disease severity. Furthermore, the levels of S-Alb in patients with myasthenic crisis were significantly lower compared with those without myasthenic crisis (Fig. 1B). As shown in Fig. 2, S-Alb was positively correlated with uric acid (*r* = 0.421, *P* < 0.001), which was inversely correlated with disease activity and disease severity.

### 3.3 Lower S-Alb increases the incidence risk of MG severity

To verify whether a decreased level of S-Alb may play a causal role in the development of MG severity, we use multivariate analysis. In unadjusted model 1, compared with subjects in the T1, those subjects in the T3 had an odds ratio (OR) of 0.148 (95% CI: 0.059–0.368). Even when adjusted for age, sex, BMI, duration of disease, diabetes, hypertension, cardiopulmonary disease, globulin, WBC, Tbil, Ibil, uric acid creatinine, HDL-C, and thymus histology, the relationship between S-Alb and MG severity remained significant in T2 and T3 with OR of 0.241 (95% CI: 0.103–0.566), 0.140 (95% CI: 0.054–0.367), respectively (Table 2).

**Table 2:**
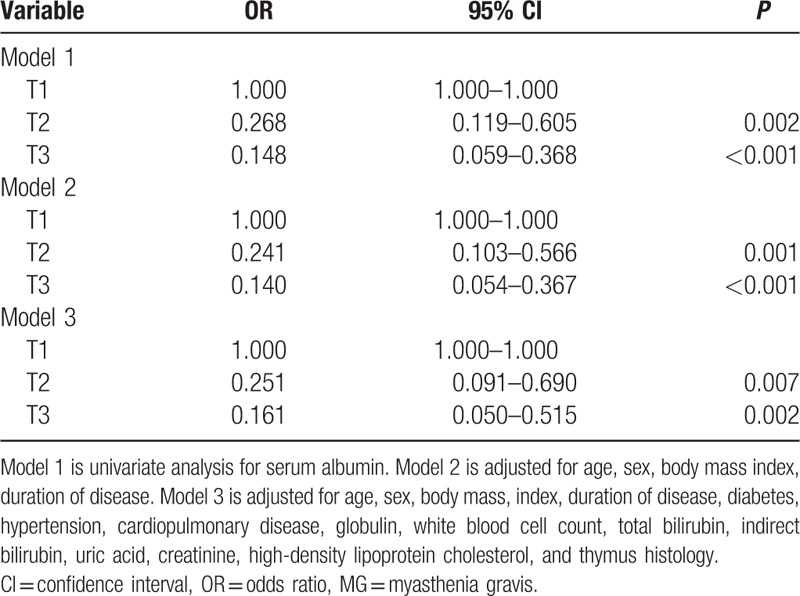
Adjusted odds ratio (95% confidence interval) for MG disability.

**Figure 3:**
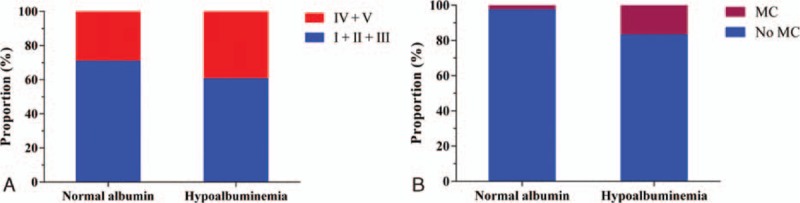
(A) The disease severity of MG in normal albumin and hypoalbuminemia groups. (B) The incidence of myasthenic crisis in normal albumin and hypoalbuminemia groups. MC = myasthenic crisis.

For the purpose of clinical application, we categorized the study population according to “Normal albumin” or “Hypoalbuminemia.” In patients from hypoalbuminemia group, 39.29% patients suffered from higher disease severity versus 21.95% from normal albumin group (*P* = 0.016). Moreover, 16.67% patients with hypoalbuminemia suffered from MC while only 2.44% patients suffered from MC in patients with normal albumin (*P* = 0.002) (Fig. 3). Furthermore, in the hypoalbuminemia group, the associations of S-Alb with MG severity remained significant (OR: 0.693, 95% CI: 0.550–0.874, *P* = 0.002) (Table 3). However, in the normal albumin group, there was no significant association between S-Alb and MG severity (OR: 0.827, 95% CI: 0.604–1.134, *P* = 0.238) (Table 3).

**Table 3:**
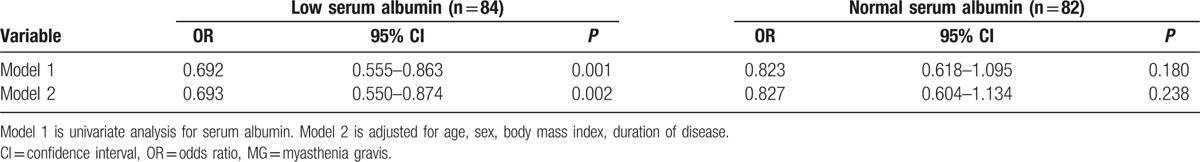
Multivariate linear regression analysis of the relationship between serum albumin and MG disability in low serum albumin group and normal serum albumin group, respectively.

## 4 Discussion

In this study, we analyzed the data of MG patients in a single center to investigate the association between S-Alb and the severity of disease of MG. Our data demonstrated that hypoalbuminemia frequently occurred in MG patients; the lowest level of S-Alb was associated with the severity of disease of MG as well.

MG, which is antibody-mediated, T cell-dependent, and complement involved, is a severe autoimmune disease characterized by loss of AChR on the postsynaptic membrane of neuromuscular junction and resulted in impaired neuromuscular transmission and muscle weakness.^[11]^ Recently, accumulating evidence has shown that oxidative stress might play a major role in the pathogenesis in MG.^[9,12,13]^ Indeed, Venkatesham et al^[14]^ were the first to report that reactive oxygen species might contribute damage toward the AChR. Additionally, Krishnaswamy and Cooper^[15]^ has suggested that the main targets of the attacks by reactive oxygen species are the highly conserved cysteine residues in nicotinic AChR.

S-Alb, a 65-kDa protein, the most abundant protein in human plasma, shows significant antiinflammatory activity.^[16,17]^ On the other hand, there are accumulating data supporting the antioxidant properties of S-Alb.^[5,18]^ Because of its reactive oxygen intermediated scavenging activity and its transition metal ion-binding activity, S-Alb represents the predominant circulating antioxidant agent in plasma exposed to continuous oxidative stress.^[19]^ S-Alb contains 1 free cysteine-derived redox-reactive thiol (–SH) group (Cys^34^), which confers a major role in serum antioxidant capacity.^[20]^ Furthermore, Lang et al^[21]^ has demonstrated S-Alb could attenuate HOCl-induced oxidative damage in vitro in a dose-dependent manner. Given that inflammation and oxidative stress are involved in the pathogenesis of MG,^[8,9,12]^ it has been postulated that S-Alb level may be associated with the development of MG. In this study, we found that the S-Alb levels reflected the severity of MG. Low S-Alb levels were significantly related to low uric acid, which is known to be associated with disease severity in MG.^[9]^

It is noteworthy that the association of S-Alb concentrations with disease severity was significant, especially in hypoalbuminemic patients. Few studies have designed to evaluate the importance of hypoalbuminemia as a predictive marker of the severity of disease of MG. To our best knowledge, the present study is the first report demonstrating the relationship between hypoalbuminemia and the severity of disease of MG. Albumin solutions have been widely employed in the intensive care to set for correction of hypoalbuminemia and intravascular volume expansion in critical patients.^[22]^ S-Alb is a marker of disease severity of MG, nevertheless, it was uncertain whether treating with hypoalbuminemia will improve the outcome of the disease. Further larger and prospective studies are necessary to confirm the hypothesis and clarify the role of S-Alb and its underlying mechanisms in patients with MG.

In conclusion, this study showed that low S-Alb levels in MG patients are associated with clinical factors indicating of increased disease severity, especially in those with hypoalbuminemia. S-Alb could be a potential biomarker for the severity of disease of MG. However, it is just a clinical phenomenon found in single center investigation; multiple hospitals should be added to further study. We addressed the critical role of S-Alb in the patients with MG; however, seldom clinical randomized controlled trial has been performed to affirm the clinical curative effects of albumin in patients with MG. Meanwhile, more laboratory experiments need to be carried out to explore the oxidative stress status and the antioxidative potential of albumin in the pathogenesis of MG.
